# The Neural Bases of Disgust for Cheese: An fMRI Study

**DOI:** 10.3389/fnhum.2016.00511

**Published:** 2016-10-17

**Authors:** Jean-Pierre Royet, David Meunier, Nicolas Torquet, Anne-Marie Mouly, Tao Jiang

**Affiliations:** ^1^Olfaction: From Coding to Memory Team, Lyon Neuroscience Research Center, CNRS UMR 5292 – INSERM U1028 – Université de Lyon 1Lyon, France; ^2^Sorbonne Universités, Université Pierre et Marie Curie, Institut de Biologie Paris Seine, UM 119, CNRS, UMR 8246, Neuroscience Paris SeineParis, France

**Keywords:** food aversion, disgust, disliking, diswanting, reward circuit, motivational salience, basal ganglia, fMRI

## Abstract

The study of food aversion in humans by the induction of illness is ethically unthinkable, and it is difficult to propose a type of food that is disgusting for everybody. However, although cheese is considered edible by most people, it can also be perceived as particularly disgusting to some individuals. As such, the perception of cheese constitutes a good model to study the cerebral processes of food disgust and aversion. In this study, we show that a higher percentage of people are disgusted by cheese than by other types of food. Functional magnetic resonance imaging then reveals that the internal and external globus pallidus and the substantia nigra belonging to the basal ganglia are more activated in participants who dislike or diswant to eat cheese (Anti) than in other participants who like to eat cheese, as revealed following stimulation with cheese odors and pictures. We suggest that the aforementioned basal ganglia structures commonly involved in reward are also involved in the aversive motivated behaviors. Our results further show that the ventral pallidum, a core structure of the reward circuit, is deactivated in Anti subjects stimulated by cheese in the wanting task, highlighting the suppression of motivation-related activation in subjects disgusted by cheese.

## Introduction

Disgust, described for the first time as a basic emotion by Darwin (1872), is characterized by a peculiar facial expression, an action of distancing oneself from an offensive object, a distinctive physiological manifestation (nausea), and a typical feeling of revulsion (Izard, 1977; Rozin and Fallon, 1987). Disgust can result from sensory factors, bad tasting food (distaste), or anticipated harmful consequences such as poisoning (toxic food), allergenic reaction, or food intolerance (Rozin and Fallon, 1987). In the latter cases, disgust may be the result of conditioned aversion, which is a tendency to avoid something previously associated with a noxious stimulus.

What happens in the brain when food produces such disgust or aversive reaction due to learning? Several studies of patients with brain injury (Sprengelmeyer et al., 1996; Gray et al., 1997; Calder et al., 2000; Adolphs et al., 2003; Suzuki et al., 2006; Sprengelmeyer, 2007) and cerebral imaging studies of healthy subjects (Phillips et al., 1997, 1998, 2004; Sprengelmeyer et al., 1998; Heining et al., 2003; Wicker et al., 2003; Fitzgerald et al., 2004) have shown involvement of the insula and basal ganglia in disgust. However, most studies have focused on recognition of facial expressions of disgust or have used disgust-inducing images or odors not related to food. Additionally, discrepant results have also been observed among studies (Adolphs et al., 1998; Gorno-Tempini et al., 2001; Schienle et al., 2002; Milders et al., 2003; Murphy et al., 2003). Studies on aversive learning in humans have been conducted using electric shock as an unconditioned stimulus (e.g., Buchel et al., 1998), as well as thermal pain (e.g., Becerra et al., 2001) and monetary loss (Breiter et al., 2001; Delgado et al., 2008), but studies on food aversion learning are rare due to ethical issues associated with the experimental induction of gastrointestinal symptoms. It is also difficult to find aversive reactions to the same type of food because each individual presents idiosyncratic reactions. However, an exception is cheese because it is perceived as being particularly disgusting by some people. As such, the perception of cheese can provide a quite favorable experimental context to study the mechanisms of food disgust and aversion and examine the supporting neural networks.

Pleasure and reward mechanisms have been established to play central roles in the control of human food intake (Kringelbach, 2004). The incentive salience model of taste and ingestive behavior (Berridge, 1996, 2009) suggests that food reward can be operationalized in an affective component termed ‘liking’ (pleasure related to the reward) and a motivational component termed ‘wanting’ (desire to obtain the reward). These two components underlie distinct neural mechanisms and networks (Berridge, 1996, 2009; Smith et al., 2009; Richard et al., 2012; Jiang et al., 2015). Reward further involves a third psychological component that is learning one’s preferences and aversions (Berridge and Robinson, 2003).

The purpose of the present study was twofold. First, we aimed to estimate the proportion of individuals who are disgusted by cheese. We conducted a survey of the French population to evaluate individual preferences for 75 foods distributed into eight categories. Second, we aimed to compare the brain activities of individuals with aversion to cheese and those who enjoy and commonly eat it using functional magnetic resonance imaging (fMRI); we will refer to these subjects as Anti and Pro, respectively. To grasp the food disgust and aversion processes, we focused our investigation on the food reward components of affective liking and motivational wanting. Participants successively performed two tasks, and we were mainly focused on the brain responses associated with the liking and wanting scores for cheese when these scores were significantly lower in Anti than in Pro subjects, that is when Anti participants, respectively, disliked and diswanted cheese. Animal as well human studies show that the reward circuit includes areas of the basal ganglia that comprise the dorsal and ventral striatum, the globus pallidus, the ventral pallidum, and the substantia nigra (SN), but also areas of midbrain such as the ventral tegmental area (VTA) (Kalivas and Nakamura, 1999; Nolte, 1999). Areas of the prefrontal lobe such as the orbitofrontal cortex and anterior insula are also bran regions described as being important for the processing of rewards and punishments (e.g., Small et al., 2001; de Araujo et al., 2003; Gottfried et al., 2003; de Araujo and Rolls, 2004; Kringelbach and Rolls, 2004; Bragulat et al., 2010; Brown et al., 2011; Liu et al., 2011; Kuhn and Gallinat, 2012; Berridge and Kringelbach, 2013). For example, the anterior insula was shown to be activated both during the emotion of disgust evoked by unpleasant odorants and during the observation of disgusted facial expressions (Wicker et al., 2003). We hypothesized that several of these areas could be differentially activated between Pro and Anti subjects in response to odors and pictures of cheese, but not in response to odors and pictures of foods other than cheese (OFood).

## Materials and Methods

### Survey on Food Preferences

One hundred and forty-five men (38.58 ± 15.36 years; range 16.03–72.45 years) and 187 women (36.07 ± 13.74 years, range 18.63–73.78) matched in age (*t*_1,330_ = 1.57, *p* > 0.05) were recruited using newspaper advertisements and fliers. Their BMIs were 24.14 ± 3.11 and 22.18 ± 3.25, respectively (*t*_1,325_ = 5.49, *p* < 0.001), a gender difference that has been previously reported in healthy 25-year-old English individuals (Abbott et al., 2007) and in healthy American 34- to 36-year-old individuals (Jackson et al., 2002). This difference can be explained by the higher body density of males than females (Deurenberg et al., 1991).

The participants completed a questionnaire in which they had to judge how much they liked 75 food items distributed into eight categories: fruit, cheese, charcuterie, fish, vegetable, meat, dessert, and various foods (**Table 1**). They used an 11-point Likert-type scale to evaluate the food items. Subjects who strongly disliked items within a category (mean rating < 3) were questioned to assess whether the rating was motivated by food intolerance, allergic reaction, cultural influence, or specific dietary habits, such as vegetarianism. The subjects were informed that we were interested in surveying food preferences in the population but were not aware of the specific aim of the study. Participation in the study was voluntary, anonymous, and confidential. The survey was conducted in accordance with the Declaration of Helsinki.

**Table 1:** List of 75 foods that were distributed into eight categories.

	Fruit	Cheese	Charcuterie	Fish	Vegetable	Meat	Dessert	Various
1	Strawberry	**Roquefort**	Boiled ham	Sardine	Pea	Beef Bourguignon	Apple pie	Sauerkraut
2	Cherry	Camembert	Cured ham	Trout	**Cucumber**	Pork chop	Rum baba	Potée
3	Coconut	Gruyère	Dry sausage	Smoked salmon	Courgette	Roast veal	Strawb. char.	Couscous
4	Grapefruit	**Parmesan**	**Farm pâté**	Bouillabaisse	**Fennel**	Coq au vin	Chocolat tart	B. Reine
5	Orange	**Tomme**	Liver pâté	Tuna	Bean	Rabbit	Chesnut cream	Pasta
6	Passion fruit	**Cheddar**	Foie gras		Leek	Steak	Mille feuille	**Pizza**
7	Apple	**Goat cheese**	Salami		Cauliflower	Boiled chicken	Chocolat cake	Quiche
8	Kiwi	Picodon	Smoked sausage		Spinach		Brownies	Chips
9	Pear	St Félicien	Merguez		Beet		Floating island	**Peanut**
10	Mango	Munster	Rillettes		Potato		Tiramisu	Green olive
11	Red currant				**Mushroom**			
12	Pineapple							

*B. Reine, Bouchée à la Reine; Strawb. char., Strawberry charlotte. The foods selected for the fMRI experiment are emboldened.*

### fMRI Experiment

#### Ethical Statement

The study was conducted in accordance with the Declaration of Helsinki. Participants were informed about the procedures used in the tasks and provided informed written consent as required by the local Institutional Review Board according to French regulations on biomedical experiments with healthy volunteers [Ethical Committee of CPP-Sud Est II (n° CPP 07-043), DGS2007-0554, December 17, 2007]. Handedness was checked by the Edinburgh Handedness Inventory (Oldfield, 1971).

#### Participants

Fifteen healthy right-handed subjects liking cheese (11 women; mean age ± SD: 27.5 ± 4.9 years; range: 22.0–36.9 years) and 15 healthy right-handed subjects hating cheese (10 women; mean age ± SD: 30.8 ± 7.6 years; range: 18.5–42.2 years) were assigned to Pro and Anti groups, respectively. Both groups of participants were matched in age (*F*_1,28_= 1.90, *p* = 0.179) and BMI [Pro: 21.9 ± 2.2 (range: 17.8–25.1); Anti: 21.0 ± 2.2 (range: 18.3–26.2); *F*_1,25_ = 1.72, *p* = 0.202]. The participants were recruited from the university community (see Supplementary Data) using an advertisement indicating that individuals who dislike and/or are unable to eat certain foods were needed; however, the subjects were not aware that they were selected on the basis of their disgust or liking of cheese. Participants in both groups were also selected due to their preferences for six OFoods.

The participants were further checked as being without known olfactory impairments, rhinal disorders (colds, active allergies, a history of nasal-sinus surgery, or asthma), pregnancy, neurological diseases, ferrous implants (e.g., pacemakers and cochlear implants), or claustrophobia. In addition, they were screened for their olfactory detection ability (odor vs. no odor) and mean breathing cycle duration. Here, included subjects achieved at least 86.7% correct responses (Pro = 96.0 ± 5.2; Anti = 99.0 ± 3.2; *F*_1,18_= 2.29, *p* = 0.148) and had a mean breathing cycle duration of 4 s/cycle (Pro = 4.66 ± 1.01; Anti = 4.16 ± 1.59; *F*_1,18_= 0.70, *p* = 0.412).

#### Stimuli

Forty odorants were used: 28 for training purposes and 12 for the fMRI scanning session. For fMRI, stimuli included six cheese varieties (blue cheese, Cheddar, goat cheese, Gruyère, Parmesan, and tomme) and six OFoods (cucumber, fennel, mushroom, pâté, peanut, and pizza) odorants whose names were also included in the questionnaire used for the survey. They were graciously supplied by Mane (Bar-sur-Loup, France), René Laurent (Le Cannet, France), and Givaudan-Roure (Lyon, France) and purchased from Sigma-Aldrich (Saint Quentin-Fallavier, France). The odorants were diluted in odorless mineral oil (Sigma Aldrich, Saint-Quentin-Fallavier, France) to a concentration of 10% in volume. For stimuli presentation, 5 ml of this solution was absorbed into compressed polypropylene filaments inside of a 100-ml white polyethylene squeeze-bottle equipped with a dropper (Fisher Scientific, Illkirch, France).

**FIGURE 1:**
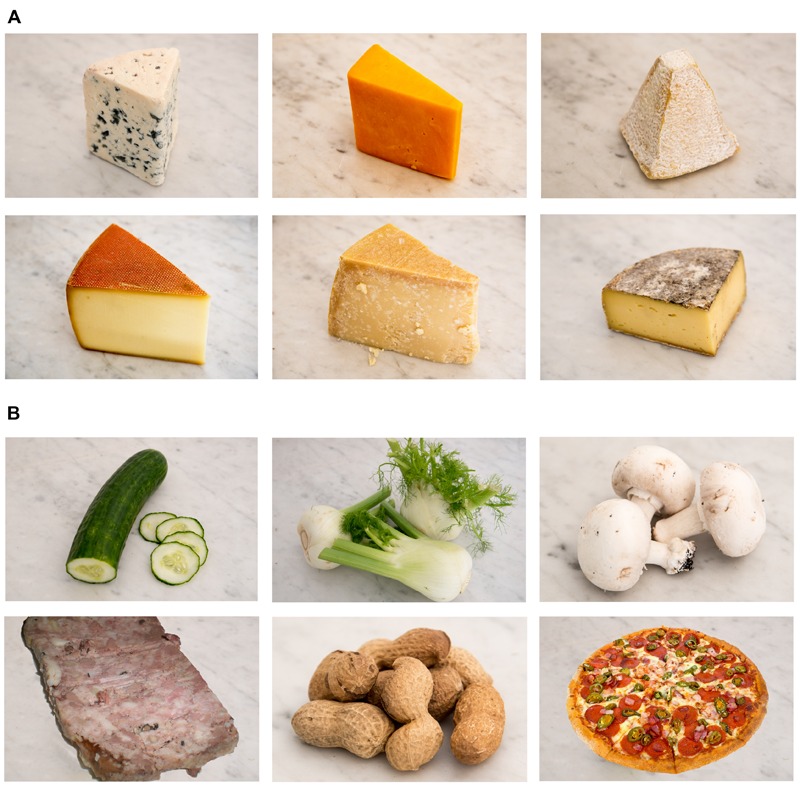
**Pictures of the two food categories. (A)** Six cheese types were used as stimuli: blue cheese, Cheddar, goat cheese, Gruyère, Parmesan, and tomme. **(B)** Six OFoods (foods other than cheese) were used as stimuli: cucumber, fennel, mushroom, pâté, peanut, and pizza.

For fMRI, 12 visual stimuli (landscape mode, 720 × 467) were selected for matching with the odor stimuli listed above (**Figure 1**). For training, six control pictures (landscape mode, 720 × 467) were computer generated, each displaying a rectangle of a different color.

#### Stimulating Materials

The odorants were presented to the participants using an airflow olfactometer, which allows the stimuli to be synchronized with breathing (Vigouroux et al., 2005). The stimulation equipment consisted of two modules: a non-ferrous (Duralumin^®^) air-dilution injection head (placed in the magnet room) and the electronic component of the olfactometer (positioned outside the magnet room). Compressed air (10 L/min) was pumped into the olfactometer and delivered continuously through a standard oxygen mask positioned on the subject’s face. At the beginning of an inspiration phase, an odorant was injected into the olfactometer by rapidly squeezing the odor bottle into the injection head, thereby transmitting the odorant to the mask. Information regarding the onset of stimulation was transmitted by optical fibers to analog-to-digital converters located outside the magnetically shielded room and powered by nickel–cadmium batteries. The presentation timing was monitored using commercially available Presentation software (Neurobehavioral Systems, Inc., Albany, CA, USA) and was synchronized with the scanner.

Participants’ responses were acquired with a five key-press button box that provided logic signals. The five buttons were placed in a configuration similar to the five fingers (thumb, forefinger, middle finger, ring finger, and pinkie) of the right hand, simulating the five levels of a Likert-type scale, respectively. Breathing was recorded using polyvinyl-chloride foot bellows (Herga Electric Limited, Suffolk, UK) secured to the subject’s abdomen with a cotton belt. The participants’ behavioral responses, breathing data, stimulation onset, and trigger signals from the MRI scanner were recorded online (100 Hz sampling rate) on a laptop equipped with a digital acquisition board I/O card (PCI-6527) (National Instruments^®^, Austin, TX, USA) using LabVIEW software package (National Instruments^®^). The data were further analyzed using custom routines created with Matlab (The Mathworks, Natick, MA, USA).

#### Experimental Design

**FIGURE 2:**
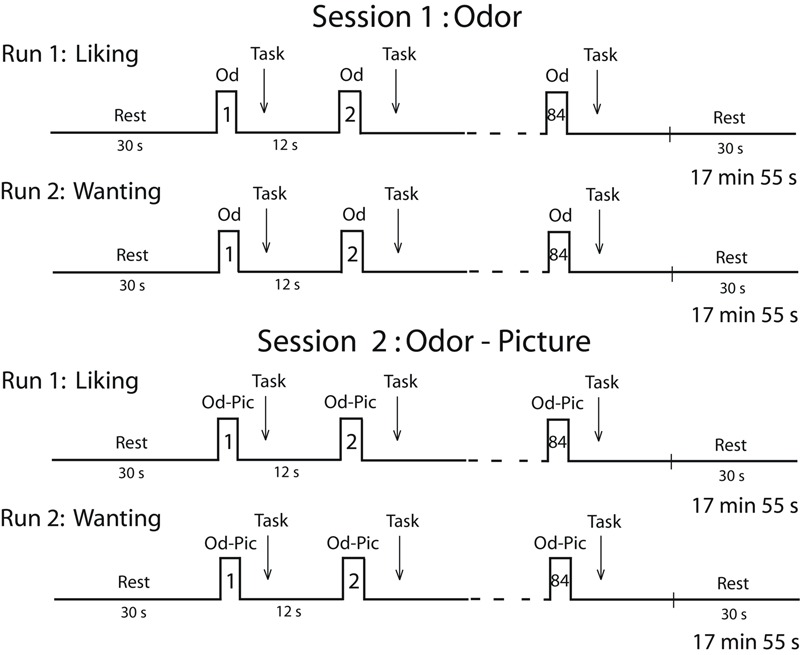
**Timeline of the procedure.** Each session was performed in the morning (during the hunger state) and included two runs during which either the liking task or the wanting task was performed. Od, odor; Od-Pic, odor and picture.

Two sessions were planned for each participant on two consecutive days (**Figure 2**). Because individuals in the survey expressed a deep disgust for the odor of cheese, and because they claimed to be able to detect cheese in a room from its odor only, we performed a first session in which subjects were stimulated in the hunger state with food odors. In a second session, to ensure that participants identified stimuli without ambiguity, we stimulated them with both odors and pictures (Od-Pic) of the same foods. During each session, two successive functional runs were performed during which the participants reported their liking and wanting of the stimuli, respectively. A structural image acquisition sequence was performed between two functional runs on the first or second day. During each run, 12 stimuli were delivered seven times each, such that 84 stimuli were presented. They were delivered according to an event-related fMRI design with a jittered inter-stimulus interval of ∼12 s, depending on the participant’s breathing. The orders of the runs were counterbalanced among the participants and the order of the presentation of stimuli was randomized for each run.

During the liking run, the participants were asked to press one of five buttons with the corresponding finger depending on their judgment (thumb: very unpleasant; forefinger: unpleasant; middle finger: neutral; ring finger: pleasant; or pinkie: very pleasant). During the wanting run, the participants were asked to press one of five buttons depending on their desire to eat the food evoked by the stimulus (not at all, not desired, just a little, much desired, or urge) at the present time. If the subject did not smell food during the first session, then they did not press a button. For each stimulus, the subjects had to provide a response as soon as they had performed their liking or wanting judgment. Subjective reward responses of liking and wanting were measured in terms of scores and response times (RT).

General instructions were provided outside the scanner. The day before receiving fMRI scans, the participants were trained outside the MR facility to breathe naturally and regularly without sniffing or holding their breath, to detect odors during inspiration while avoiding sniffing and to provide rapid finger responses using the 5-button box. They were asked to rate the intensities of 28 odorants in a first session and their familiarity in a second session by pressing one of five buttons with the corresponding finger. Control pictures (color rectangles) were synchronously presented with the odor stimuli for 3 s. On the day of fMRI scans, the subjects were specifically instructed to correctly perform the two tasks (liking, wanting) and to avoid confounding them. These instructions have been detailed in a previous study (Jiang et al., 2015). The participants wore earplugs to protect them from the scanner noise and were asked to keep their eyes open during scanning.

All participants were scanned in the hunger state (between 09:45 am and 1:30 pm) and were instructed to have a light breakfast (tea or coffee, plus a slice of bread) no later than 7:00 or 9:00 am, depending on the time at which the scan began. As the metabolic state has been shown to influence liking and wanting performances (Jiang et al., 2008), the participants’ hunger state was evaluated at the onset and end of each fMRI session using a 10-point Likert-type scale (1 = no hunger at all; 10 = extremely hungry).

#### Behavioral and Physiological Data Analysis

ANOVAs with repeated measurements (Winer et al., 1991) were used to separately analyze the scores and RTs derived from the liking and wanting tasks. Differences between pairs or groups of means were assessed using multiple orthogonal contrasts. As disgusting/aversive stimuli induce an arousal reaction related to whether or not they are toxic or not and must be avoided/rejected (Royet and Plailly, 2004) we hypothesized that Anti subjects would answer more quickly than Pro subjects because they disliked or diswanted cheese. We calculated correlation coefficients between the variables in Anti and Pro for cheese and OFood. As the comparisons were planned, no Bonferroni correction was applied (Perneger, 1998; Armstrong, 2014). To compare the scores between the liking and wanting tasks for cheese, and as these tasks are correlated (Jiang et al., 2008, 2013), we performed MANOVA using group (Anti vs. Pro) with repeated measurements on task (liking *vs*. wanting) and stimuli (six varieties of cheese) factors (Winer et al., 1991).

As breathing variations are known to impact brain activation (Sobel et al., 1998), the amplitudes of inspiratory and expiratory waveforms were estimated by integrating each of the curves located on both sides of the baseline. We analyzed the amplitudes of the two inspiratory cycles (numbered 0 and 1) following each odor stimulation and the cycle preceding the stimulation (-1). Mean cycle amplitudes were computed for the different experimental conditions: food (cheese, OFood), task, inspiration (-1, 0, 1) and group, and 3-way ANOVA (food × inspi × group) with repeated measurements was performed. Statistical analyses were performed using Statistica (StatSoft^®^, Tulsa, OK, USA).

#### Functional and Structural Data Acquisition and Preprocessing

Images were acquired using a 1.5-Tesla MAGNETOM Sonata whole-body imager (Siemens Medical^®^, Erlangen, Germany) equipped with a 4-channel circularly polarized head coil. For functional imaging, we obtained 26 interleaved, 4-mm-thick axial slices using a T2^∗^-weighted echo-planar sequence with the following parameters: repetition time (TR) = 2500 ms, echo time (TE) = 50 ms, flip angle (FA) = 80°, field-of-view (FOV) = 240 mm × 240 mm, and imaging matrix = 64 × 64 (voxel size: 3.75 mm × 3.75 mm × 4 mm). In total, 390 scans were collected for each functional run. A high-resolution structural T1-weighted anatomical image (inversion-recovery 3D Gradient-Echo sequence, 1 mm × 1 mm × 1 mm) parallel to the bicommissural plane and covering the entire brain was acquired over ∼10 min. Foam wedges were used to restrict head motion. An oil-filled capsule was fixed on the right temple to subsequently locate the right side of the images.

For each subject, the first five volumes of each functional run were discarded to avoid T2^∗^ non-equilibration effects. We then processed all functional images using a pipeline in Nipype workspace (Gorgolewski et al., 2011) that provided a neuroimaging data processing framework in Python by implementation of Statistical Parametric Mapping software (SPM8, Wellcome Department of Cognitive Neurology, London, UK, Friston et al., 1995b). Slices of each remaining volume were slice-timing-corrected. All functional volumes were realigned to the median volume, co-registered to the anatomical image, spatially normalized to the Montréal Neurological Institute (MNI) standard brain T1.nii of 2 mm × 2 mm × 2 mm (Friston et al., 1995a), and smoothed with an 7.5 × 7.5 × 8-mm full-width half-maximum Gaussian kernel that is considered to be optimal for both single-subject inference and for group inference in statistical parametric maps (Mikl et al., 2008). No participant moved more than 3 mm in any direction within or across runs. Thus, no data were eliminated due to motion artifacts.

#### Functional Data Analyses

Preprocessed data were statistically analyzed on a subject-by-subject basis using the General Linear Model implemented in SPM8. For each subject, activation associated with three factors of interest [food (cheese, OFood), task (liking, wanting), and modality (Odor, Od-Pic)] was modeled as events (corresponding to the onset times for each condition) convolved with both the canonical hrf and its time derivative (Friston et al., 1998; Hopfinger et al., 2000). A high-pass filter (cut-off frequency of 1/120 Hz) was used to eliminate instrumental and physiological signal fluctuations at very low frequencies. As the hrf varies depending on the subject and area of interest (Handwerker et al., 2004), we attempted to better estimate this function using both the canonical hrf and its time derivative (Hopfinger et al., 2000). Because of uncertainty in the onset of sniffing the odor, we used the amplitude of the hrf in group random-effect analysis, which removes potential bias in results caused by latency (Calhoun et al., 2004). Stimulus onset asynchronies were fixed at the time of odor delivery. Confounding factors (head motion) were included in the model. Random-effects analyses were performed to extrapolate statistical inferences at the population level, as described in the SPM8 software. Whole-brain analyses were performed on functional images for the different experimental conditions. We then contrasted activation functional images obtained for these two tasks between Pro and Anti [Pro vs. Anti] for either cheese or OFood. For these analyses, the level of significance was set at *p* < 0.005, uncorrected at the cluster level for multiple comparisons across the much larger volume of the whole brain. We used an extent threshold (*k*) superior or equal to five adjacent activated voxels. To check whether areas classically activated in studies related to disgust, (but not observed here using the above contrasts) could be revealed using further analysis, we extracted activation in four conditions (cheese, food, liking, wanting) for the Od-Pic stimuli and performed a conjunction (intersection) analysis of the simple contrasts [Cheese – Baseline] and [OFood – Baseline] during liking and wanting (contrast [Cheese – Baseline] ∩ [OFood – Baseline]). Baseline activity was extracted from periods of rest recorded at the beginning and at the end of each run (30 s each), and also from between trials periods. For the conjunction analysis, the level of significance was set at *p* < 0.05 using family-wise error correction for multiple comparisons over the entire brain and an extent threshold of *k* superior or equal to 10 adjacent activated voxels. The anatomical atlases edited by Duvernoy (1999) and Mai et al. (2008) were used to identify activated regions.

As a dichotomy has been suggested to exist between the ventral and dorsal striatum (Mawlawi et al., 2001; Delgado et al., 2008), we completed previous analyses by examining functional activation in several Volumes-Of-Interest (VOIs) of the striatum (the ventral and dorsal parts of the caudate nucleus and the putamen and anterior and posterior parts of the NAc). To distinguish among these different structures, we first selected these regions using the Harvard–Oxford probabilistic atlas^1^ developed by Kennedy et al. Second, precise delineations were determined based on the visual differentiation of structures (T1 images) from the MNI template (Ch2bet.nii) using MRIcron^2^, detailed diagrams and pictures from the human brain atlas created by Mai et al. (2008), and the boundary criteria proposed by Mawlawi et al. (2001). The delineations were drawn from coronal slices of the caudate nucleus (from *y* = 20 mm anterior to *y* = -27 mm posterior to the anterior commissure), the anterior and posterior parts of the NAc (from *y* = 20 to 14 and from *y* = 13 to 6, respectively), and the dorsal and ventral parts of the putamen (from *y* = 17 to -20 and from *y* = 24 to 10, respectively). The mean activation signals were bilaterally extracted under two experimental conditions (food, task) for each subregion of the VOIs and for each of the 30 participants using the python package nibabel^3^. Five-way ANOVA with repeated measures and multiple orthogonal comparisons were then performed to compare the levels of activation as a function of the different experimental conditions [group (Anti vs. Pro), task (liking vs. wanting), food (cheese vs. OFood), area (ventral vs. dorsal), and side (right vs. left] for the Od-Pic stimuli only.

Brain linear regression analyses were further performed to evaluate whether the activation data were correlated with the self-rated liking/wanting data. This assessment was performed for the structures of striatum and midbrain that, according to our hypotheses, were differentially activated between Anti and Pro: the internal and external segments of the globus pallidus (GPi/GPe), the SN and VTA.

## Results

### Survey on Food Preferences

**FIGURE 3:**
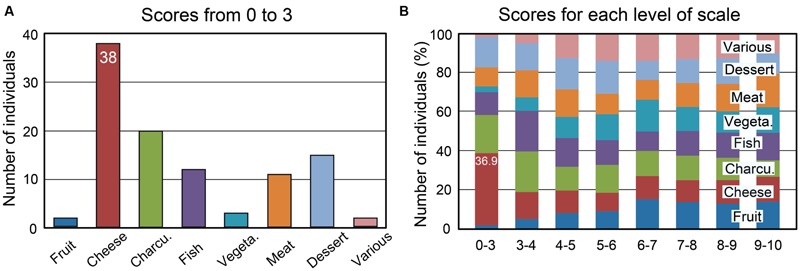
**Number of individuals among 332 as a function of the mean liking scores and the eight food categories. (A)** Number of individuals with mean liking scores of 0 to 3. **(B)** Percentages of individuals as a function of the mean scores for each of the eight food categories. Charcu., charcuterie; Vegeta., vegetable.

Percentages of individuals as a function of the rating scale scores (from 0 to 10) were computed for the eight food categories and are illustrated in Supplementary Figure 1. We found that the proportion of individuals disliking cheese was higher compared with other food categories. Twenty out of the 332 tested individuals (6%) rated cheese with a score of 0 or 1. The scores from 0 to 3, which can be considered to represent disgust, were observed in 38 individuals (11.5%) (**Figure 3A**). The percentages of individuals as a function of the liking scores for the eight food categories are shown in **Figure 3B** for each level of the scale. Individuals disgusted by cheese represented 36.9% of those with a score of 0 to 3 for the eight food categories. Most of these individuals claimed to be disgusted by the odor and taste of cheese (60%), while 18% reported a cheese intolerance or allergy. Forty-seven percent of 38 individuals indicated that at least one family member also disliked cheese. This familial particularity was found to affect up to six family members, including ascendants and descendants. Three individuals did not eat cheese due to hypercholesterolemia; therefore, we estimated that the total proportion of individuals disliking cheese was 10.5%. Concerning the other foods (charcuterie, fish, and meat), the low scores were mainly related to the individuals’ cultural origin or specific dietary habits, such as vegetarianism.

### fMRI Experiment

#### Behavioral and Physiological Data

**FIGURE 4:**
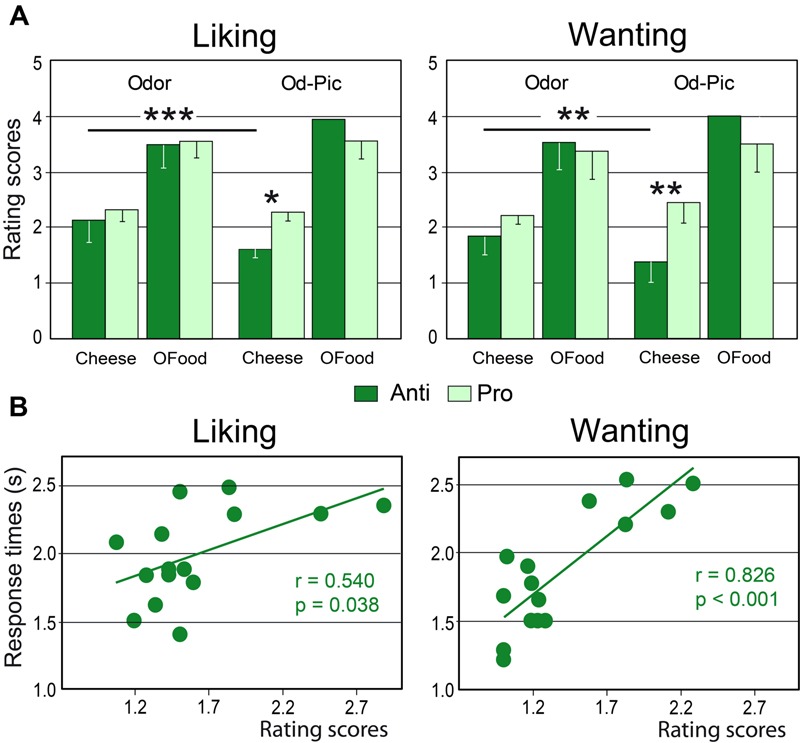
**Behavioral performances. (A)** Mean liking and wanting scores for cheese and OFood presented as odor or Od-Pic stimuli to Anti and Pro subjects. **(B)** Positive correlations between the rating scores and the response times recorded in the two tasks in Anti subjects exposed to cheese (Od-Pic) stimuli. OFood, other foods than cheese; Od-Pic, odor plus picture. Error bars, standard deviations. ^∗^*p* < 0.05, ^∗∗^*p* < 0.01, ^∗∗∗^*p* < 0.001.

Mean liking and wanting scores were determined for cheese and OFood (food factor) in both groups of subjects exposed to 12 odor or Od-Pic (modality factor) stimuli (**Figure 4A**). For each task, four-way (modality × food × stimuli × group) analysis of variance (ANOVA) mainly revealed a significant food effect due to lower scores for cheese than for OFood (liking: *F*_1,28_= 154.7, *p* < 0.001; wanting: *F*_1,28_= 129.6, *p* < 0.001; these significant differences are not indicated by an asterisk in **Figure 4A**), and a significant modality × food × group interaction (liking: *F*_1,28_= 13.93, *p*’s < 0.001; wanting*: F*_1,28_= 13.84, *p* < 0.001), indicating that scores for cheese were significantly lower for the Od-Pic stimuli in Anti than in Pro (liking: *p* = 0.032; wanting: *p* = 0.004) and were significantly lower than those for odor stimuli (*p*’s ≤ 0.003). As liking and wanting behavioral scores for cheese did not significantly differ between Anti and Pro subjects stimulated only with odors (liking: *p* = 0.517; wanting: *p* = 0.290), we focused the next analyses on the Od-Pic stimulus condition.

Comparing rating scores for cheese between the liking and wanting tasks, a multivariate ANOVA (MANOVA) then revealed a marginally significant group × task interaction (Roy’s *GR*_5,52_= 2.32, *p* = 0.056) suggesting that reduction in scores in Anti compared with Pro was significantly larger for the wanting than for the liking task. This result suggests that the subjects may have used different cognitive processes during cheese evaluation.

Analysis of RTs for cheese and OFood during both tasks in both groups of subjects further revealed a significant food × group interaction (*F*_1,28_= 6.48, *p* = 0.036), mainly due to the shorter RTs to cheese in Anti than in Pro (*p* < 0.036). Furthermore, the RTs to cheese in Anti were significantly correlated with the scores during the two tasks (liking: *r* = 0.540, *F*_1,13_ = 5.36, *p* = 0.038; wanting: *r* = 0.826, *F*_1,13_ = 28.01, *p* < 0.001; **Figure 4B**); the lower the score, the lower the RT. No other RTs in Pro and Anti were significantly correlated with scores (0.027 < *r* < 0.425, *p*’s ≥ 0.114).

We next examined whether differences in liking and wanting rating scores between Anti and Pro could be attributed to varying hunger states. A two-way (time × group) ANOVA with repeated measurements showed a significant increase in the hunger state from the onset to the end of the session (time factor: *F*_1,23_= 77.76, *p* < 0.001), but no significant difference between the two groups of subjects, and no significant interaction between factors were noted.

**FIGURE 5:**
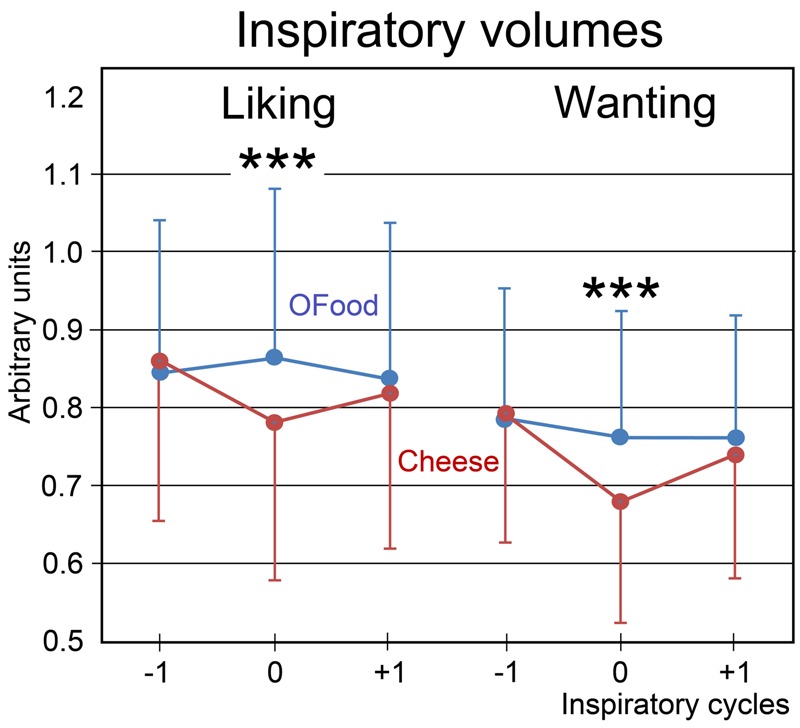
**Variations in the inspiratory volumes.** Volumes during the liking and wanting tasks are shown for Anti subjects exposed to Od-Pic stimuli of either cheese (in black) or OFood (in white). Error bars, standard errors of the mean (SEM); ^∗∗∗^*p* < 0.001.

Finally, we investigated the impact of the stimuli on the amplitude of inspiratory (inspi) volumes. No significant effect of the group factor was noted (*F*_1,28_ = 0.24, *p* = 0.626). A four-way (food × task × inspi × group) ANOVA with repeated measurements revealed a significant interaction between the four factors (*F*_2,56_ = 3.97, *p* = 0.025). This interaction indicated that inspiratory volumes in Anti (**Figure 5**) during both liking and wanting tasks were significantly smaller (9.5 and 11.4%, respectively) during stimulations (inspi 0) with cheese than during those with OFood (*p*’s < 0.001), but not during the previous (-1) and the next (+1) inspiratory cycles. It further showed in Pro a smaller inspiratory volume for cheese than OFood during the wanting task (7%, *p* = 0.002).

#### fMRI Data

**FIGURE 6:**
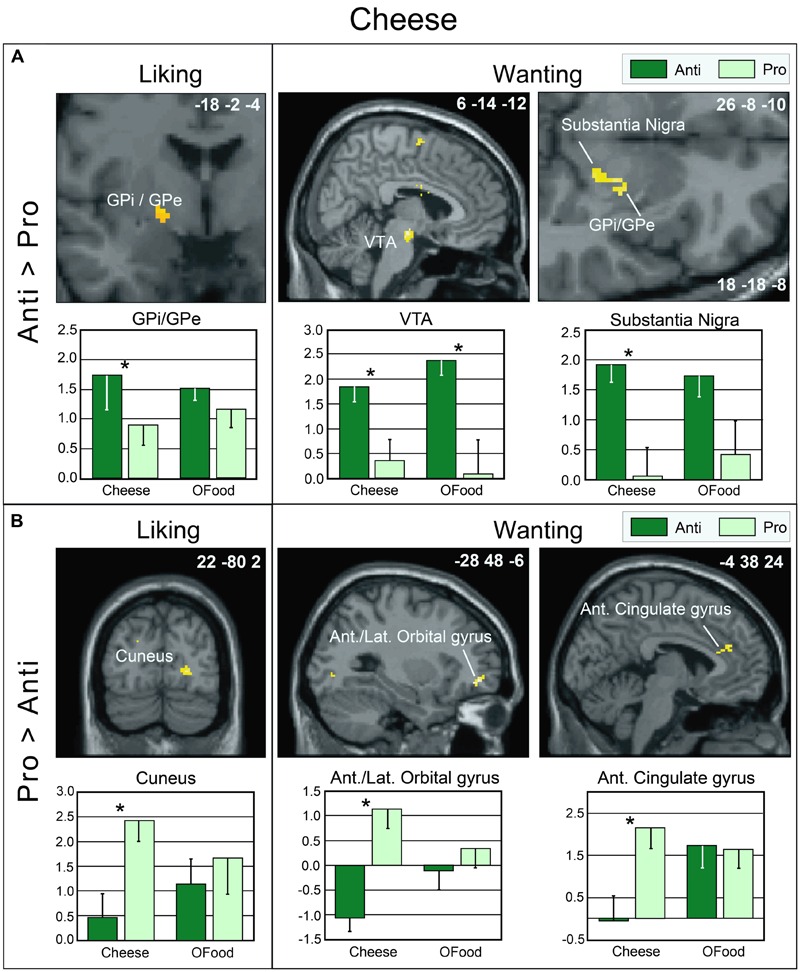
**Brain sections showing differentially activated regions in Pro and Anti subjects exposed to cheese Od-Pic stimuli during the liking and wanting tasks. (A)** Higher activation in Anti than in Pro. **(B)** Higher activation in Pro than in Anti. Bar graphs show the levels of activation in Anti and Pro for cheese and OFood. The mean levels of activation for OFood were extracted from the same clusters as those found for cheese and are given by comparison. Ant., Anterior; Lat., lateral; GPi/GPe, internal and external segments of globus pallidus; VTA, ventral tegmental area; error bars, SEM; ^∗^*p* < 0.05.

As mentioned above, no significant differences were observed between the rating scores of the participants in the two groups stimulated with odors only. Therefore, we examined the brain imaging data obtained with the Od-Pic stimuli. We first investigated whether neural networks were differentially activated between Anti and Pro participants ([Anti – Pro] and [Pro – Anti] contrasts) exposed to cheese or OFood stimuli during the liking and wanting tasks (**Figures 6A,B**). For cheese stimuli, we found a higher activation of the GPi/GPe during liking, and of the VTA, GPi/GPe and SN during wanting in Anti compared with Pro (**Table 2**; **Figure 6A**). For OFood stimuli, we observed a higher activation of the VTA in Anti than Pro during wanting (Supplementary Table S1).

**Table 2:** Brain areas differentially activated in Pro and Anti subjects exposed to cheese Od-Pic stimuli.

Task	Contrast	Brain areas	*k*	*T*	*x*	*y*	*z*
LIKING	Anti > Pro	Postcentral gyrus	136	5.35	64	-34	40
		Superior frontal gyrus	78	4.21	8	-4	66
		Supramarginal gyrus	43	4.11	-60	-52	40
		Middle frontal gyrus	30	4.08	-44	38	34
		Middle frontal gyrus	36	3.83	42	-4	62
		Middle frontal gyrus	27	3.56	-36	-6	66
		GPi/GPe	19	3.18	-18	-2	-4
	Pro > Anti	Anterior cingulate gyrus	32	4.60	20	44	4
		Cuneus	34	3.99	22	-80	2
		Lingual gyrus	56	3.67	32	-66	-4
WANTING	Anti > Pro	Cerebellum	35	4.26	-10	-84	-18
		Supramarginal gyrus	38	4.02	-58	-40	50
		Superior frontal gyrus	62	3.93	12	-2	66
		VTA	37	3.93	6	-14	-12
		GPi/GPe	35	3.63	26	-8	-10
		SN		3.35	18	-18	-8
	Pro > Anti	Ant./Lat. orbital gyrus	38	4.79	-28	48	-6
		Anterior cingulate gyrus	32	3.81	-4	38	24

*Ant./Lat., Anterior/Lateral; SN, substantia nigra; GPi/GPe, internal and external segments of globus pallidus; VTA, ventral tegmental area; k, size of cluster in number of connected voxels; T, Student’s t value; x, y, z, MNI coordinates (in mm) of the maximum peak.*

**FIGURE 7:**
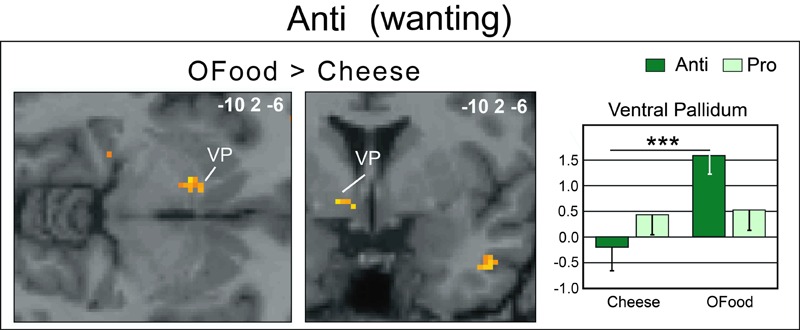
**Horizontal and coronal brain sections showing more activation of the VP in Anti subjects stimulated with OFood than cheese Od-Pic stimuli during the wanting task.** The bar graph shows the levels of activation in Anti and Pro for cheese and OFood. The mean levels of activation for cheese and OFood in Pro were also extracted in the VP by comparison. VP, ventral pallidum; error bars, SEM; ^∗∗∗^*p* < 0.001.

Second, we investigated whether the brain areas with increased activation by cheese in Anti compared with Pro (**Table 2**; **Figure 6A**) exhibited similar activation in Anti when we compared the activation images for cheese and OFood. We performed the contrast [Cheese – OFood] during the liking and wanting tasks (Supplementary Table S2), but did not detect activation in any reward circuit areas, such as VTA, GPi/GPe or SN, in Anti participants. However, the inverse contrast [OFood – Cheese] revealed significant activation of the left VP (**Figure 7**; Supplementary Table S2), a small area posteriorly and ventrally located at the anterior commissure, and in areas involved in odor perception (posterior orbital gyrus) and memory (parahippocampal gyrus, hippocampus). No critical area of the basal ganglia or midbrain was found to be differentially activated by OFood and cheese in Pro subjects (Supplementary Table S3).

We further observed that in Pro, the liking and wanting scores for OFood were negatively correlated with activation of the GPi/GPe (-18 -2 -4) (liking: *r* = -0.568, *F*_1,13_= 6.21, *p* = 0.027; wanting: *r* = -0.724, *F*_1,13_= 14.28, *p* = 0.002); thus, the lower the liking and wanting scores for OFood, the higher the activation of the GPi/GPe. This result is consistent with the high activation observed in Anti subjects for cheese. No other significant correlations were found in the GPi/GPe, VTA or SN (*r*’s ≤-0.477, *F*’s_1,13_*≤* 3.83, *p*’s ≥ 0.072).

The insula is commonly investigated in studies on disgust (Phan et al., 2002; Murphy et al., 2003; Wicker et al., 2003; Fitzgerald et al., 2004; Calder et al., 2007; Mataix-Cols et al., 2008). As we did not observe any activation in this region in contrast analyses, we checked whether it was activated under the four experimental conditions (cheese-liking, cheese-wanting, OFood-liking, OFood-wanting) in Anti and Pro subjects exposed to Od-Pic stimuli. Conjunction analysis revealed strong bilateral activation in the insula (Supplementary Table S4). Thus, although this region was activated under all experimental conditions, it did not exhibit more activation in Anti disgusted by cheese than in Pro.

As the striatum has been involved in aversive learning (Delgado et al., 2008) and functional organization of the striatum distinguishes the ventral and dorsal parts of the caudate nucleus and putamen (Mawlawi et al., 2001), we further explored functional activation of these areas by performing VOIs. As functional differences between the rostral and caudal parts of the NAc have been observed in rats (Ho and Berridge, 2014), we also subdivided the NAc into anterior and posterior areas, but did not detect any significant differences in activation of these areas between Anti and Pro.

## Discussion

In humans, it is difficult to find disgust reactions to the same type of food because each of us has acquired idiosyncratic reactions. Moreover, food aversion has been under-researched because it is ethically inconceivable to experimentally induce illness in humans. In this study, we showed that a substantial proportion of people in France are disgusted by cheese and that this situation is experimentally favorable for studying the cerebral processes of food disgust and aversion. In these individuals, cheese odors and pictures induce stronger activation of the GPi/GPe, and SN than in people who like and eat cheese. This finding suggests that the GPi/GPe and SN code the hedonic (not only positive but also negative) and motivational components of food reward. Further, we observed that the lack of desire to eat cheese (diswanting) is associated with lack of activation of the VP, a core structure in incentive motivation (Smith et al., 2009). Thus motivation-related activation is suppressed in Anti subjects disgusted by cheese.

### Survey

To assess whether disgust for cheese is widespread among individuals, we performed a survey of the French population. It revealed that among the individuals showing disgust for a given food, those disliking cheese represented a higher proportion (6% with a score of 0 to 1 on an 11-point scale) than those disliking the other food categories. This finding is rather surprising because France is the country with the greatest variety of cheeses (Sperat-Czar and Boulenger^4^) and one of the countries with the highest levels of cheese consumption. It suggests that similar results might be observed in other countries with similarly high levels of cheese consumption, such as western European countries and the United States. Sixty percent of the individuals disliking cheese expressed disgust/aversion for cheese in all forms (odor, visual aspects, and texture). It was not possible to distinguish whether aversive reactions resulted from a simple disgust or a conditioned aversion. Eighteen percent of the subjects stated that they had milk intolerance, a generic term that includes lactose intolerance (Wilson, 2005). The symptoms of lactose intolerance were first described by Hippocrates (Chobot, 1951) but are today recognized and diagnosed medically; they include gastrointestinal symptoms (e.g., abdominal pain, nausea, and vomiting) and debilitating systemic symptoms (e.g., headache and allergy) (Matthews et al., 2005). Pharmacological food intolerance has also been reported and is caused by substances (e.g., histamine and tyramine) present in fermented foods, such as cheese (Ortolani and Vighi, 1995). Ingestion of food containing one or more of these amines can result in toxic symptoms in individuals with a low susceptibility threshold. In brief, food intolerance is particularly prone to induce food aversion, a type of conditioning initially described by Pavlov (1927), then extensively studied in animal, but also in human (Barker et al., 1977; Milgram et al., 1977). In such a situation, aversive reaction of subjects for cheese would be more related to expected negative consequences than to disgust for its sensory properties.

Lactose intolerance involves dairy products that contain lactose, but semi-soft and hard cheeses (e.g., cantal, cheddar, and raclette) no longer contain lactose after processing. Therefore, disgust reactions observed for these cheeses in individuals with lactose intolerance are likely caused by a generalization effect. In the absence of identified intolerance (e.g., due to genetic predisposition), disgust for cheese shared by several members of the same family begs the question of whether it results from a simple social transmission, is in sum vicariously acquired, or is the consequence of an epigenetic determinism.

### Behavioral and Physiological Data

We observed that the liking and wanting scores were globally lower for cheese than for OFood in both Anti and Pro. This result was surprising and suggested that the appetitive properties of the odors and pictures were decreased for cheese compared with OFood. However, focusing on cheese data, we found that the liking and wanting scores were lower in Anti than in Pro subjects stimulated with both odors and pictures, but not with odors only. The responses of the subjects to the odors alone could have been partly influenced by an inability to easily identify cheese odors, as they are fundamentally difficult to name/identify (Olofsson et al., 2014). This interpretation further means that the odor emotional impact, which has been reported to predict memory (Herz and Cupchik, 1992, 1995) and identification (Bestgen et al., 2015) performances, is here insufficient to trigger disgust reaction to cheese in Anti subjects. These findings demonstrate that the identification more than sensory characteristics of disgusting odors is important to induce an appropriate response. In other words, “it’s the subject’s conception of the object rather than the sensory properties of the object that primarily determines the hedonic value” (Rozin and Fallon, 1987). In addition to systematically reported disgust at the time of selection, Anti subjects selected here for fMRI clearly manifested this disgust by a decreased inspiratory volume (>10%) during Od-Pic stimulation. They blocked their breathing to avoid smelling cheese odors. This result may be related to the closing of the nares that is characteristic of the expressions of disgust (Rozin and Fallon, 1987), but also to changes in heart rate and skin conductance autonomic responses (Stark et al., 2005). A small reduction in inspiratory volume for cheese during the wanting task in Pro subjects suggests that other processes may also play a role.

### Functional Data

During the liking task, marginally higher activation of the GPi/GPe was detected in Anti than in Pro subjects stimulated with cheese but not with OFood. During the wanting task, differential activation of the GPi/GPe, SN and VTA between the groups was also observed for cheese. However, as the VTA was also differentially activated between the groups for OFood, only the SN and GP were considered critical areas with more specific involvement in Anti than in Pro for cheese. We also found that the VP was significantly less activated in Anti subjects stimulated with cheese than with OFood.

The aforementioned structures belong to the reward circuit and are commonly involved in appetitive situations. For instance, Schur et al. (2009) have observed that the VTA is involved in reward processing and is selectively attuned to representations of foods perceived as fattening. Joshua et al. (2009) have demonstrated that GPi and GPe single units in monkey are more strongly modulated by and better reflect the probability of reward- than aversive-related events. Here, we consistently noted that the lower the liking and wanting scores for OFood, the higher the activation of the globus pallidus.

Although neuroimaging studies on the neural substrates of disgust have mainly focused on the insula, caudate and putamen (Phillips et al., 1997, 1998, 2004; Sprengelmeyer et al., 1998; Heining et al., 2003; Wicker et al., 2003; Fitzgerald et al., 2004), the globus pallidus, also known as the dorsal pallidum, has recently drawn attention (Murphy et al., 2003; Calder et al., 2007; Mataix-Cols et al., 2008). Calder et al. (2007) have demonstrated that disgust sensitivity (scale) is significantly correlated with activation of the pallidum (-14 -2 -6) in response to the presentation of pictures of disgusting, but not appetizing or bland, foods. In the present study, activation of the GPi/GPe was increased in Anti compared with Pro subjects during the liking and wanting tasks, while the respective scores were lower in Anti subjects. By hypothesizing that the liking and wanting rates are the inverse reflections of the disgust scores (i.e., negatively correlated), our results are consistent with those of Calder et al. (2007) and Mataix-Cols et al. (2008).

How can we explain the exacerbated response of GPi/GPe and SN to cheese in Anti subjects? The projections from the GPe to the GPi and SN pars reticularis, two major output structures of the basal ganglia, are well established (Kita, 2007; Nambu, 2007). Can these structures that code for incentive salience wanting also be involved in cheese rejection? Berridge and Robinson (1998) and Berridge (2007) have argued that the dopaminergic system may be involved in both reward and aversively motivated behaviors. To satisfy both alternatives, they have suggested the terms “*motivational salience*” rather than “*incentive salience.*” To explain how a positive process such as wanting can be involved in aversive situations, Gray (1975) presented the example of individuals in a fearful situation who may ‘want’ to escape to a safe place or to perform another response that gains safety. Other interpretations have further been proposed, for example that the two types of salience are mediated by different dopamine neurons. Recently, Ikemoto et al. (2015) have reported that most neurons of the ventral and dorsal striatum are medium-size spiny neurons that can be classified into two types of neurons with complementary activities: neurons that express D_1_ receptors and those that express D_2_ receptors. Consistently, Kravitz et al. (2012) have shown that optogenetic excitation of D_1_-neurons in the striatum is rewarding, whereas that of D_2_-neurons is aversive. Given that the GPi and SN are two major output structures of the basal ganglia, we can suggest that such a double system is also present in these structures.

An important result of our study is the lack of activation of the VP in Anti subjects exposed to cheese stimuli during the wanting task. Several studies have suggested that the VP could represent “an essential convergent point for hedonic and motivational signaling pathways in the brain” (Smith et al., 2009). For example, subjects deploy more physical force to earn higher amounts of money and pallidal activity is then linearly related to the amount of force produced (Pessiglione et al., 2007). This result was observed even when money stakes were presented too quickly to be consciously detected. Motivation-related activation of the VP has also been observed when food odors predict immediate arrival of their associated drink (Small et al., 2008), when high-calorie food images are presented to obese women (Stoeckel et al., 2008), or still when subjects in the hunger state are stimulated by food odors (Jiang et al., 2015). Thus, as the VP was here less activated (and even deactivated) by cheese than by OFood in Anti subjects, our results are consistent with those of these studies. We therefore propose that motivation-related activation was suppressed in Anti subjects disgusted by cheese. Furthermore, the lack of differential response between cheese and OFood in the VP in Pro suggests that the hedonic and motivational impacts of the two types of food were presumably similar.

The VP occupies a prominent place in the circuit mediating the integration of reward perception and adaptive behavioral responses (Kalivas and Nakamura, 1999). It integrates GABAergic, glutamatergic, dopaminergic, and opioid signals from the NAc, striatum, amygdala, and prefrontal cortex (Olive and Maidment, 1998; Kalivas and Nakamura, 1999; Kelley, 1999; Zahm, 2000; Tindell et al., 2006). In rat, inactivation of the VP with muscimol (a GABA_A_ receptor agonist) produces excessive disgust reactions to sweet tastes (Shimura et al., 2006), whereas its activation by bicuculline (a GABA_A_ receptor antagonist) decreases aversion to a taste previously paired with gastric malaise (Inui et al., 2007). It is reported that the core region responsible for ‘disgust’ (also reported in terms of hedonic hotspot) is limited to the posterior half of the VP (Smith et al., 2009; Ho and Berridge, 2014; Berridge and Kringelbach, 2015). Thus, whereas an increase in opioid transmission in the posterior VP enhances hedonic ‘liking’ reactions to sucrose as well as motivational ‘wanting’ to eat (Smith et al., 2009), damage to this site by excitotoxic lesions or temporary inactivation causes excessive disgust reactions to sucrose (Ho and Berridge, 2014). However, in human, it is very difficult to distinguish between the anterior and posterior parts of the VP due to its small size and low spatial resolution of fMRI. Therefore, it is unknown whether the deactivation of the VP observed herein during cheese-induced disgust can be compared to posterior VP lesions/inactivation in rat and the resulting intense sensory disgust. Whether this deactivation is the consequence of a removed descending inhibitory control is also a yet unsolved question that deserves further investigation.

## Conclusion

The study of food disgust and aversion in humans is difficult because it is rare to find individuals who present disgust for the same type of food, and it is not conceivable to experimentally induce a food aversion by provoking gastro-intestinal symptoms. Our findings show that a higher-than-expected proportion of individuals are disgusted by cheese than by other food categories. These individuals dislike cheese to the point that they cannot eat it, an experimental context quite adapted to studying the brain mechanisms of food disgust. Odor and sight of cheese activate the GPi/GPe and SN, indicating that in addition to encoding reward, these structures may also encode disgust and thus the aversive properties of food. We also report that motivation-related activation of the VP in response to food is suppressed in individuals disgusted by the smell and/or sight of cheese. In brief, our findings show that disgust for cheese, which may be the result from an initial physiological discomfort, is associated with modified activation of the mesocorticolimbic circuitry of reward.

## Author Contributions

J-PR planned and designed research. NT and J-PR performed the experiments. DM and J-PR analyzed data. J-PR drafted manuscript and prepared figures. J-PR, DM, NT, A-MM and TJ edited and approved final version of manuscript.

## Conflict of Interest Statement

The authors declare that the research was conducted in the absence of any commercial or financial relationships that could be construed as a potential conflict of interest.

## Footnotes

1 http://neuro.debian.net/pkgs/fsl-harvard-oxford-atlases.html

2 http://people.cas.sc.edu/rorden/mricron/index.html

3 http://nipy.org/nibabel/

4 http://www.socheese.fr
