# Supplementary material for: The Neural Bases of Disgust for Cheese: An fMRI Study
Source: Front Hum Neurosci. 2016 Oct 17;10:511. doi: 10.3389/fnhum.2016.00511 (PMC5065955; doi:10.3389/fnhum.2016.00511)
Supplement: Supplementary file 1 [file Table_1.PDF]

Table S1. The table indicates the brain areas that were differentially activated in Pro and Anti subjects during the liking and wanting tasks when the subjects were stimulated with Od-Pic stimuli of OFoods**.**

Task	Contrast	Brain areas	*k*	*T*	*x*	*y*	*z*
LIKING	Anti > Pro	Cerebellum	71	5.84	-2	-50	-26
		Supramarginal gyrus	48	4.67	46	-30	36
		Precentral gyrus	94	4.48	40	-8	66
		Middle frontal gyrus	59	4.28	-34	-4	66
		Angular gyrus	74	4.27	-14	-66	46
		Putamen	24	3.86	26	-4	4
		Superior frontal gyrus	162	3.84	16	2	70
		Supramarginal gyrus	27	3.64	64	-32	40
	Pro > Anti	Anterior cingulate gyrus	53	4.13	16	42	8
		Inferior occipital gyrus	108	3.62	32	-70	-2
WANTING	Anti > Pro	Superior parietal gyrus	28	4.59	18	-84	32
		Precentral gyrus	34	4.45	-32	-12	42
		VTA	41	4.38	4	-14	-16
		Supramarginal gyrus	58	4.26	-26	-48	34
		Parahippocampal gyrus	31	4.04	-20	-18	-20
		Hippocampus			-28	-10	-22
		Supramarginal gyrus	52	3.90	-50	-42	42
		Cerebellum	76	3.84	-2	-66	-18
	Pro > Anti	Fusiform gyrus	35	4.20	-40	-62	-12
		Superior frontal gyrus	51	4.00	-8	50	22
		Lingual gyrus	37	3.71	24	-56	-8

VTA, ventral tegmental area; k, size of the cluster in number of connected voxels; T, Student’s t value; x, y, z, MNI coordinates (in mm) of the maximum peak.
