# Supplementary material for: The Neural Bases of Disgust for Cheese: An fMRI Study
Source: Front Hum Neurosci. 2016 Oct 17;10:511. doi: 10.3389/fnhum.2016.00511 (PMC5065955; doi:10.3389/fnhum.2016.00511)
Supplement: Supplementary file 2 [file Table_2.PDF]

Table S2. The table indicates the brain areas that were differentially activated in Anti subjects during the liking and wanting tasks when the subjects were stimulated with either cheese or OFood stimuli ([Cheese – OFood] and [OFood – Cheese] contrasts).

Task	Contrast	Brain areas	k	T	x	y	z
LIKING	Cheese -OFood	Middle frontal gyrus	19	5.04	−42	52	20
		Superior occipital gyrus	22	4.76	28	-74	28
		Cingulate gyrus	165	4.71	18	−50	4
		Striate area (17)	129	4.69	−10	−64	4
		Inferior frontal gyrus	47	4.64	50	30	2
		Cingulate gyrus	15	4.49	6	-22	36
		Precuneus	68	4.47	−10	−80	36
		Precentral gyrus	17	4.35	52	−10	42
		Superior occipital gyrus	13	4.17	14	-82	24
		Middle temporal gyrus	33	4.11	38	−74	8
		Cuneus	42	3.85	4	-82	16
	OFood - Cheese	Precentral gyrus	54	5.66	−4	−28	68
		Middle occipital gyrus	438	5.44	−24	−100	−4
		Middle frontal gyrus	37	4.94	28	6	44
		Cuneus	405	4.79	12	−82	−2
		Anterior cingulate gyrus	43	4.76	−10	44	−4
		Lateral orbital gyrus	21	4.42	-44	44	-10
		Middle frontal gyrus	56	4.32	−38	4	42
		Angular gyrus	32	4.33	42	-70	32
		Middle frontal gyrus	28	4.00	40	−12	66
		Postcentral gyrus	46	3.85	−42	−26	62
WANTING	Cheese - OFood	Precuneus	90	4.83	10	−74	34
		Inferior lingual gyrus	183	4.28	26	−54	6
	OFood - Cheese	Superior occipital gyrus	1696	6.74	−16	−102	0
		Middle frontal gyrus	49	6.31	−32	16	50
		Lateral /Posterior orbital gyrus	63	6.23	−44	38	−12
		Superior frontal gyrus	42	5.78	16	52	10
		Angular gyrus	428	5.68	−44	−68	28
		Gyrus rectus	234	5.16	−2	44	−18
		Anterior cingulate gyrus	296	5.06	−4	36	12
		Middle temporal gyrus	149	5.03	-58	2	-24
		Posterior cingulate gyrus	97	4.98	−6	−46	34
		Superior frontal gyrus	13	4.95	−14	56	34
		Superior frontal gyrus	52	4.78	−14	38	54
		Middle temporal gyrus	148	4.67	-58	-60	-4
		Precentral gyrus	64	4.67	−24	−48	72
		Parahippocampal gyrus	115	4.62	−24	−28	−16
		Superior parietal gyrus	62	4.54	−32	−60	58
		Angular gyrus	20	4.51	−46	−56	30
		Superior parietal gyrus	41	4.48	−28	−44	50
		Ventral pallidum	14	4.32	-8	0	-8
				4.00	-10	0	-4
				3.42	-14	6	-8
				3.28	-8	6	-6
		Superior frontal gyrus	42	4.31	−16	68	8
		Posterior cingulate gyrus	57	4.31	4	-50	22
		Middle frontal gyrus	33	4.31	−28	28	46
		Precentral gyrus	167	4.25	−40	−28	54
		Middle temporal gyrus	66	4.24	50	−46	−8
		Postcentral gyrus	100	4.19	−44	−28	18

Amygdala	68	4.18	-28	2	-22
Middle occipital gyrus	38	4.16	46	-54	20
Angular gyrus	48	4.15	38	-68	38
Inferior lingual gyrus	48	4.16	32	−66	−14
Precuneus	44	4.08	2	-62	20
Anterior pulvinar	26	4.07	16	-28	10
Hippocampus	26	4.02	30	-6	-16
Inferior temporal gyrus	27	3.91	46	0	−32
Cingulate gyrus	129	3.90	14	−24	48
Lateral pulvinar nucleus	31	3.88	−22	−30	8
Parahippocampal gyrus	25	3.84	26	−26	−18

k, size of the cluster in number of connected voxels; T, Student's t value; x, y, z, MNI coordinates (in mm) of the maximum peak
