# Supplementary material for: The Neural Bases of Disgust for Cheese: An fMRI Study
Source: Front Hum Neurosci. 2016 Oct 17;10:511. doi: 10.3389/fnhum.2016.00511 (PMC5065955; doi:10.3389/fnhum.2016.00511)
Supplement: Supplementary file 3 [file Table_3.PDF]

Table S3. The table includes the brain areas that were differentially activated in Pro subjects during the liking and wanting tasks when the subjects were stimulated with cheese or OFood stimuli.

Task	Contrast	Brain areas	k	T	x	y	z
LIKING	Cheese -OFood	Cuneus	102	4.61	−12	−82	6
		Cerebellum	13	4.58	-6	-56	-30
		Cuneus	12	4.49	12	−78	8
		Lingual gyrus	32	4.30	-24	-64	4
		Cuneus	15	4.25	20	-84	12
		Supramarginal gyrus	22	4.11	44	-34	38
		Middle occipital gyrus	15	3.92	−42	−70	−6
		Postcentral gyrus	30	3.91	56	4	8
		Precentral	59	3.87	46	4	36
		Superior frontal gyrus	16	3.80	18	42	36
		Posterior orbital gyrus	20	3.67	44	36	-12
		Supramarginal gyrus	25	3.65	-46	-36	50
		Superior parietal gyrus	21	3.60	32	-56	64
		Inferior frontal gyrus	37	3.52	-44	22	4
	OFood - Cheese	Angular gyrus	426	5.35	−38	−72	32
		Middle frontal gyrus	44	4.92	−38	12	50
		Precuneus	125	4.74	−6	−58	34
		Cuneus	76	4.52	12	−86	−6
		Superior frontal gyrus	106	4.35	−6	64	−4
		Superior frontal gyrus	17	4.29	−22	34	56
		PC / Antero-ventral insula	45	4.25	−30	0	−16
		Superior frontal syrus	30	4.19	-8	52	42
		Cuneus	43	4.01	26	−78	−10
		Middle temporal gyrus	55	3.96	-58	-2	-18
		Middle frontal gyrus	14	3.95	-38	6	52
		Middle temporal gyrus	22	3.89	-64	-52	0
		Superior occipital gyrus	48	3.87	−22	−100	−2
		Fusiform gyrus	22	3.86	−50	−60	−18
		Middle temporal gyrus	41	3.86	-48	-28	-6
		Superior frontal gyrus	18	3.78	-4	48	30
WANTING	Cheese -OFood	Superior temporal gyrus	13	5.23	−42	14	−22
		Precuneus	289	5.00	−4	−76	24
		Postcentral/Supramarginal gyrus	16	4.99	−30	−46	38
		Superior parietal gyrus	153	4.80	6	−78	48
		Middle frontal gyrus	23	4.77	42	14	44
		Middle occipital gyrus	20	4.36	46	−62	−8
	OFood - Cheese	Superior lingual gyrus	511	6.19	−22	−76	−10
		Medial orbital gyrus	183	4.91	−14	48	−10
		Angular gyrus	24	4.80	−44	−80	28
		ParaHippocampal gyrus	15	4.68	26	−24	−18
		Lingual gyrus	134	4.60	14	−72	−8
		Postcentral gyrus	29	4.40	46	−22	20
		Posterior orbital gyrus	36	4.36	−30	30	−18
		Posterior cingulate gyrus	111	4.32	−8	−46	48
		Superior parietal gyrus	60	4.12	-22	-58	62
		Anterior pulvinar gyrus	24	3.84	-18	-28	4
		Anterior cingulate gyrus	71	3.74	-2	52	2

k, size of the cluster in number of connected voxels; T, Student's t value; x, y, z, MNI coordinates (in mm) of the maximum peak.
