# Supplementary material for: The Neural Bases of Disgust for Cheese: An fMRI Study
Source: Front Hum Neurosci. 2016 Oct 17;10:511. doi: 10.3389/fnhum.2016.00511 (PMC5065955; doi:10.3389/fnhum.2016.00511)
Supplement: Supplementary file 4 [file Table_4.PDF]

**Table S4.** The table shows that the results of conjunction analysis between the four experimental conditions (cheese-liking, cheese-wanting, OFood-liking, OFood wanting) in Pro and Anti subjects stimulated with Od-Pic stimuli.

| Brain areas | *k* | *T* | *x* | *y* | *Z* |
|---|---|---|---|---|---|
| Fusiform gyrus | 7381 | 12.55 | 34 | −50 | -22 |
| Superior frontal gyrus | 285 | 10.37 | -4 | 16 | 48 |
| Insula | 430 | 9.12 | -40 | 2 | 0 |
| Pulvinar | 104 | 8.67 | 20 | -28 | -2 |
| Postcentral gyrus | 708 | 7.99 | -54 | -24 | 50 |
| Insula | 240 | 7.42 | 40 | 12 | -2 |
| Pulvinar | 52 | 7.08 | -20 | -24 | -2 |
| Precentral gyrus | 30 | 6.56 | -56 | 10 | 30 |
| Postcentral gyrus | 34 | 6.46 | 34 | -54 | 54 |
| Superior parietal gyrus | 11 | 6.35 | 14 | -70 | 42 |

k, size of the cluster in number of connected voxels; T, Student's t value; x, y, z, MNI coordinates (in mm) of the maximum peak
