# Supplementary material for: The Neural Bases of Disgust for Cheese: An fMRI Study
Source: Front Hum Neurosci. 2016 Oct 17;10:511. doi: 10.3389/fnhum.2016.00511 (PMC5065955; doi:10.3389/fnhum.2016.00511)
Supplement: Supplementary file 5 [file Data_Sheet_1.PDF]

1 Supplementary Data

1.1 fMRI experiment

1.1.1 Participants

Several inclusion criteria for Pro and Anti participants were needed for fMRI experiment. The participants had to be between 20 and 35 years of age, right-handed, and available for several days, and they had to satisfy specific medical conditions. Further, because Anti and Pro subjects had to be very contrasted in their liking for cheese, both had to like other foods that were selected for the fMRI experiment. As the number of individuals disliking cheese in the survey on food preferences was insufficient to satisfy these conditions, we performed another selection of subjects using the same questionnaire and the same procedure but indicated that we were searching for individuals who disliked or who were unable to eat certain foods.

From the 72 individuals who responded to the advertisement, we observed that the proportion of those disliking cheese was much higher in the fMRI experiment than in the survey. Mean scores were approximately 25.0% and 44.4% when the subjects responded on average from 0 to 1 or from 0 to 3, respectively. Of the 72 individuals, 15 were assigned to the Anti group, and 15 were assigned to the Pro group. Their mean liking responses that were calculated from responses given for 6 cheeses and 6 OFoods were 7.61 ± 1.27 and 7.16 ± 0.816 for the Pro group, respectively, and 0.39 ± 0.60 and 6.76 ± 0.94 for the Anti group, respectively. A two-way ANOVA showed a significant group x food interaction ($F_{1, 28} = 364.5$, $p < 0.0001$), indicating a score in the Anti group that was significantly lower than that in the Pro group for cheese ($p < 0.0001$) and significantly lower than that in both Anti and Pro groups for OFoods (*p's* < 0.0001).
