# Supplementary material for: The Neural Bases of Disgust for Cheese: An fMRI Study
Source: Front Hum Neurosci. 2016 Oct 17;10:511. doi: 10.3389/fnhum.2016.00511 (PMC5065955; doi:10.3389/fnhum.2016.00511)
Supplement: Supplementary file 6 [file Image_1.PDF]

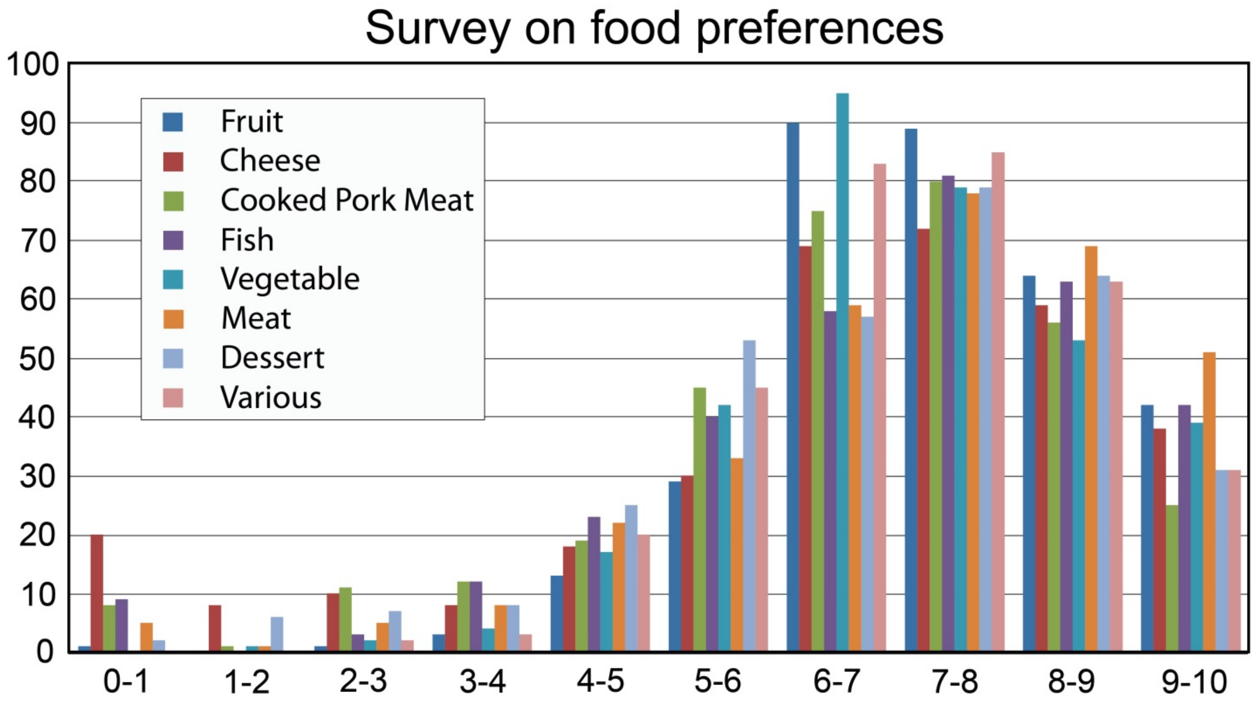


**Supplementary Figure 1.** It displays distributions of the proportion of individuals as a function of the liking rating scale (from 0 to 10) and the 8 categories of food. We compared distributions between food categories from a statistical point of view. Because several values were too small for the first three levels of the liking scale, we grouped the data with scores from 0 to 3 and found that the distributions significantly differed between the categories of food ($\chi^2_{49} = 149.0$, $P < 0.0001$). By further considering data of the first three levels of the scale only (but by discarding fruit, vegetable and various food categories for which the number of responses was too small), we also found that the 5 remaining distributions were significantly different ($\chi^2_{49} = 19.5$, $P < 0.013$).
